# 泽布替尼联合利妥昔单抗治疗伴CD79B及MYD88突变的原发皮肤弥漫大B细胞淋巴瘤，腿型1例

**DOI:** 10.3760/cma.j.issn.0253-2727.2023.03.015

**Published:** 2023-03

**Authors:** 光彩 钟, 晓宁 于, 杰 李, 春燕 陈

**Affiliations:** 1 山东大学齐鲁医院血液科，济南 250012 Department of Hematology, Qilu Hospital of Shandong University, Jinan 250012, China; 2 山东大学齐鲁医院老年医学科，济南 250012 Department of Geriatric Medicine, Qilu Hospital of Shandong University, Jinan 250012, China

患者，男，90岁，冠心病30余年，高血压10余年，因“右下肢及右腹壁多发结节2个月余”于2021年7月入院。右下肢活检病理：（右小腿）弥漫大B细胞淋巴瘤，非生发中心型；免疫组化：CD20（+），CD79a（+），Bcl-2（+），CD2（−），CD3（−），CD10（−），MUM-1（−），c-Myc（−），CD30（−），ALK（−），EMA（−），CD56（−），S-100（−），HMB45（−），TIA-1（−），Bc1-6（−），Ki-67阳性率80％，原位杂交：EBER（−）。查体：神志清，精神可，不能自行站立，查体合作。全身浅表淋巴结未触及，肝脾肋下未及，右下前腹壁可见两个突起性结节，右下肢可见红棕色无痛性皮损，高出皮面，大小约10 cm×5 cm，表面轻微渗出。右下肢凹陷性水肿，双下肢肌力Ⅳ级，肌张力正常。血常规、肝肾功能未见异常，红细胞沉降率48 mm/1 h，C反应蛋白11.1 g/L，LDH 276 U/L，EB病毒DNA未检出。动态心电图提示心律失常（频发房性早搏，短阵性房性心动过速，多源性室性心动过速，完全性右束支传导阻滞）。PET-CT示右侧小腿皮下水肿，右小腿中下部肌层/间隙内软组织结节/肿块及右侧小腿、左中侧腹壁及右下前腹壁多部位皮肤、皮下病灶及上述区域淋巴结高度摄取FDG，结合病理，提示为活动性淋巴瘤。腿部皮肤分泌物培养示金黄色葡萄球菌、阴沟肠杆菌。诊断为原发皮肤弥漫大B细胞淋巴瘤，腿型（非生发中心型，non-GCB），T2aN2M0，IPI评分3分，中高危，ECOG评分3分。根据药敏结果给予左氧氟沙星抗感染治疗，于2021年7月予利妥昔单抗700 mg单药治疗，肿块未见明显缩小。2021年8月完善二代测序（NGS）检测：CD79B、MYD88、PIM1、SPEN突变，免疫组化：PD-L1阳性率约85％，周围少数免疫细胞阳性。考虑利妥昔单抗单药效果不佳，结合NGS结果给予ZR方案（利妥昔单抗700 mg第1天；泽布替尼160 mg口服每日2次）化疗，化疗间期规律口服泽布替尼160 mg每日2次。给予第7周期ZR方案时，入院心电图提示心房颤动，后自行转复，动态心电图较前无明显变化，考虑泽布替尼的心脏不良反应，第8周期泽布替尼减量至80 mg每日2次。2～8个周期ZR方案治疗期间患者右侧小腿水肿消退，包块消失，皮肤无隆起，可见圆形色素沉着，部分融合，无皮肤破损，无渗液。2022年3月复查PET-CT示右侧小腿中下部肌层/间隙内软组织病灶未见明显显示；右侧小腿、左中侧腹壁及右下前腹壁等处FDG代谢活性明显降低；右侧盆壁及右侧腹股沟区淋巴结体积缩小、FDG代谢活性减低；Deauvile评分2分。患者目前泽布替尼80 mg每日2次口服维持治疗，处于完全缓解状态。

